# Diurnal Triglyceridemia in Relation to Alcohol Intake in Men

**DOI:** 10.3390/nu5125114

**Published:** 2013-12-16

**Authors:** Ana Torres do Rego, Boudewijn Klop, Erwin Birnie, Jan Willem F. Elte, Victoria Cachofeiro Ramos, Luis A. Alvarez-Sala Walther, Manuel Castro Cabezas

**Affiliations:** 1 Department of Internal Medicine, Center for Diabetes and Vascular Medicine, St Franciscus Gasthuis, P.O. Box 10900, Rotterdam 3004 BA, The Netherlands; E-Mails: atorresd@salud.madrid.org (A.T.R.); boudewijn.klop@gmail.com (B.K.); jwfelte@planet.nl (J.W.F.E.); 2 IiSGM (Instituto de Investigación Sanitaria), HGU Gregorio Marañón (Hospital General Universitario Gregorio Marañón), Madrid 28007, Spain; E-Mail: lalvarezsalaw@gmail.com; 3 Department of Statistics and Education, St Franciscus Gasthuis, P.O. Box 10900, Rotterdam 3004 BA, The Netherlands; E-Mail: e.birnie@sfg.nl; 4 Institute of Health Care Policy and Management, Erasmus University Rotterdam, Rotterdam 3004 BA, The Netherlands; 5 Department of Physiology, School of Medicine, Universidad Complutense de Madrid, Madrid 28007, Spain; E-Mail: vcara@ucm.es; 6 Department of Medicine, School of Medicine, Universidad Complutense de Madrid, Madrid 28007, Spain

**Keywords:** ethanol, lipemia, postprandial, triglyceride

## Abstract

Fasting and postprandial triglyceride concentrations largely depend on dietary and lifestyle factors. Alcohol intake is associated with triglycerides, but the effect of alcohol on diurnal triglyceridemia in a free living situation is unknown. During three days, 139 men (range: 18–80 years) measured their own capillary triglyceride (cTG) concentrations daily on six fixed time-points before and after meals, and the total daily alcohol intake was recorded. The impact of daily alcohol intake (none; low, <10 g/day; moderate, 10–30 g/day; high, >30 g/day) on diurnal triglyceridemia was analyzed by the incremental area under the cTG curve (∆cTG-AUC) reflecting the mean of the six different time-points. Fasting cTG were similar between the alcohol groups, but a trend of increased cTG was observed in men with moderate and high alcohol intake after dinner and at bedtime (*p* for trend <0.001) which persisted after adjustment for age, smoking and body mass index. The ∆cTG-AUC was significantly lower in males with low alcohol intake (3.0 ± 1.9 mmol·h/L) (*n* = 27) compared to males with no (7.0 ± 1.8 mmol·h/L) (*n* = 34), moderate (6.5 ± 1.8 mmol·h/L) (*n* = 54) or high alcohol intake (7.2 ± 2.2 mmol·h/L) (*n* = 24), when adjusted for age, smoking and body mass index (adjusted *p* value < 0.05). In males, low alcohol intake was associated with decreased diurnal triglyceridemia, whereas moderate and high alcohol intake was associated with increased triglycerides after dinner and at bed time.

## 1. Introduction

Many factors determine fasting and postprandial plasma triglycerides (TG) [1] like gender, age, physical activity, body mass index, dietary habits and genetic factors [2,3,4,5]. There is substantial evidence that high alcohol intake has been associated with an increase in TG and subsequent increased risk for the development of pancreatitis [6]. However, the association between alcohol and TG is complex and does not always fit a simple linear association since a J-shaped association between alcohol intake and TG has recently been described [7,8]. This reported J-shaped association is similar to the J-shaped association between alcohol intake and cardiovascular risk [9,10,11,12,13]. These epidemiologic studies suggest that low amounts of alcohol intake may lower TG concentrations. However, evidence concerning the effect of low to moderate alcohol intake in relation to TG is inconsistent. Decreased, unchanged and increased TG have all been described in relation to small-to moderate alcohol intake [12,14,15,16,17,18], whereas high alcohol intake has been consistently related to elevated TG [15,19]. Differences may exist between the effects of alcohol on fasting and postprandial TG since a postprandial increase in alcohol-mediated free fatty acid oxidation may exaggerate postprandial triglyceridemia while fasting TG remained within normal ranges [20].

The effect of alcohol consumption on diurnal triglyceridemia has not yet been studied and data on the impact of alcohol on both fasting and non-fasting TG in an uncontrolled real life situation are limited. In this observational study we investigated the impact of alcohol consumption on capillary TG concentrations (cTG) during the day determined in free living male subjects, using a hand-held point-of-care testing device [2,21,22,23,24,25].

## 2. Experimental Section

### 2.1. Subjects

Subjects were healthy male volunteers or patients, aged from 18 to 80 years, with hyperlipidemia, a known medical history of cardiovascular disease (CVD) or type 2 diabetes mellitus (T2DM) participating in several clinical studies with the same protocol, aimed at investigating factors influencing postprandial lipemia [2,21,22,23,24,25]. Exclusion criteria were the presence of renal, liver or thyroid disease. Subjects using lipid lowering drugs were investigated after withdrawal of these drugs during four weeks. Height, weight, body mass index (BMI) and blood pressure were measured at inclusion. The study was approved by the Local Medical Ethical Committee of the UMC Utrecht (University Medical Center Utrecht, Utrecht, The Netherlands). The study protocol has been registered at Clinicaltrials.gov (NCT01786421).

### 2.2. Analytical Determinations

Fasting blood samples were collected at inclusion after a period of 12 h fasting for measurement of lipids, apolipoproteins, insulin and glucose from plasma. Cholesterol, plasma triglycerides (TG) and high density lipoprotein (HDL) cholesterol (obtained after precipitation with dextransulphate/MgCl_2_), were determined using a Vitros 250 analyzer (Johnson & Johnson, Rochester, NY, USA). Plasma apolipoprotein (apo) B was measured by nephelometry using apo B monoclonal antibodies (Behring Diagnostics NV, OSAN 14/15, Marburg, Germany). Plasma apo AI was measured by nephelometry using apo AI monoclonal antibodies (Behring Diagnostics NV, OUED 14/15). Plasma glucose was measured by glucose oxidase dry chemistry (Vitros GLU slides, Johnson & Johnson, Clinical Diagnostics, Rochester, NY, USA) and colorimetry, and insulin was measured by competitive radio immunoassay with polyclonal antibodies. Low density lipoprotein (LDL) cholesterol was calculated using the Friedewald formula.

### 2.3. TG Self-Measurements

Self-measurements of cTG were performed with a hand-held point-of-care testing device (Accutrend GCT^®^, Roche Diagnostics, Mannheim, Germany). Subjects were instructed to carefully wash and dry their hands thoroughly before each measurement. With a lancing device, a drop of blood (30 μL) from the finger was obtained, which was applied to the TG test strip in the TG analyzer. The subjects recorded the diurnal TG profile in a diary. They were instructed to measure their cTG for three days (preferably on Monday, Wednesday and Friday) on the following fixed time-points: fasting, before lunch, 3 h after lunch, before dinner, 3 h after dinner and at bedtime. Subjects were requested to avoid heavy physical activity, although normal daily activities were allowed. The measurement range for cTG was 0.80–6.86 mmol/L. The TG-analyser is known to measure TG reliably, regardless of the nature of the TG-carrying lipoprotein species. The correlation coefficient between cTG measurements with the TG-analyser and plasma measurements according to enzymatic methods is 0.94 [26]. Similar results have been obtained in our laboratory [2,21]. Capillary TG concentrations are generally 0.2–0.3 mmol/L higher than TG concentrations in venous plasma [25], which may reflect differences between capillary and venous samples or differences in methodology.

### 2.4. Dietary Intake

Dietary intake on the days of cTG measurements was recorded in a diary. All answered questionnaires were evaluated individually by a trained physician together with each subject. Quantities of daily intake were estimated according to guidelines given by a dietician. Foods consumed were converted into nutrients with a version of the Dutch Nutrient Data Base [27]. Alcohol consumption of less than 10 g/day was considered low, 10–30 g/day was considered moderate and more than 30 g/day was considered high, as described previously [28]. The exact time of alcohol consumption during the day was not recorded. Subjects did not receive any suggestion concerning the composition and frequency of their meals and were requested to use their regular diet and alcohol drinking pattern during the study.

### 2.5. Statistics

Data were obtained from different studies using the same protocol. Data are given as mean ± SD unless stated otherwise. Alcohol intake was divided into: none, low (<10 g/day), moderate (10–30 g/day) and high (>30 g/day). Differences between groups were tested by ANOVA with Bonferroni as *post hoc* analysis test, Chi-square test or Fisher’s exact test where appropriate. ANCOVA was used to test for significant differences in cTG between alcohol groups at the six independent time-points adjusted for age, body mass index (BMI) and smoking. Diurnal triglyceridemia was defined as the incremental area under the curve (∆cTG-AUC). Dietary intake and diurnal triglyceridemia were calculated using the average over two or three days. Linear regression analysis was used to test for significant differences in diurnal triglyceridemia between the different alcohol groups with and without adjustment for age, BMI and smoking, since BMI closely correlates with TG and alcohol intake interacts with both smoking and TG [29]. Logarithmic transformation was performed for plasma and capillary TG in order to obtain a normal distribution, but untransformed values are shown in the text and tables. All statistical analyses were performed using PASW statistics version 18.0 (SPSS Inc., Chicago, IL, USA). The ∆cTG-AUC was calculated with PRISM version 3.0 (Graph Pad Software, San Diego, CA, USA). Statistical significance was set at *p* < 0.05 (two-sided).

## 3. Results

General characteristics of the 139 male subjects are shown in Table 1. The clinical characteristics according to alcohol intake are shown in Table 2. Men with high alcohol intake were significantly older compared to men who did not consume alcohol. Men with high alcohol consumption were more frequently smokers compared to men with less alcohol intake. The BMI tended to be lower in men with low alcohol intake (*p* for trend 0.07). No differences were found in fasting plasma TG between males with different amounts of alcohol consumption. Total energy intake was similar between alcohol groups (Table 2).

**Figure 1:**
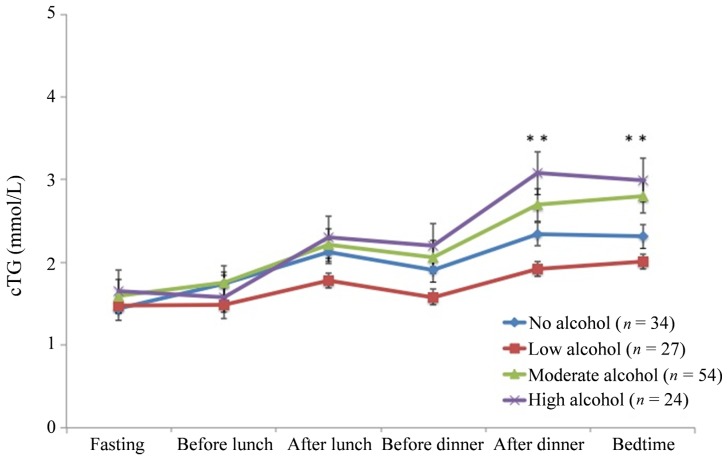
Diurnal capillary triglyceride patterns according to alcohol intake. Data are shown as mean ± SEM. ** Significant differences in cTG for a specific time-point (*p* for trend <0.001).

Patterns for diurnal capillary triglyceridemia according to alcohol intake are shown in Figure 1. No significant differences were found in fasting cTG and ∆cTG-AUC within the different groups between the three individual measurement days. A pronounced diurnal cTG increase ranging from 0.40 ± 1.00 to 1.20 ± 0.99 mmol/L was observed in all four groups. No differences in cTG between the alcohol groups were observed at time-points fasting until before dinner neither in the univariate analysis nor, after adjustment for age, BMI and smoking. However, the cTG differed significantly between the alcohol groups after dinner (*p* for trend <0.001) and at bedtime (*p* for trend <0.001), which persisted after adjustment for age, BMI and smoking (*p* = 0.036 and *p* = 0.006, respectively) (Figure 1). Low alcohol intake was associated with decreased cTG, whereas moderate and high alcohol intake was associated with increased cTG after dinner and at bedtime.

No significant differences were found for the ∆cTG-AUC between no, low, moderate and high alcohol intake in the univariate analysis (Table 2). However, after adjustment for age, smoking and BMI, men with low alcohol intake had a significantly lower ∆cTG-AUC compared to men with no, moderate or high alcohol intake (adjusted *p* < 0.05) (Table 2).

**Table 1:** Clinical and biochemical characteristics of the 139 participants to the study.

Characteristic	(*n* = 139)
Age (years)	39.9 ± 14.2
BMI (kg/m^2^)	24.6 ± 3.4
SBP (mmHg)	125.0 ± 13.5
DBP (mmHg)	81.5 ± 10.2
Smoking (*n*, %)	35 (25.2%)
History of CAD (*n*, %)	27 (19.4%)
History of T2DM (*n*, %)	3 (2.2%)
Total cholesterol (mmol/L)	5.30 ± 1.04
LDL-C (mmol/L)	3.45 ± 1.00
HDL-C (mmol/L)	1.23 ± 0.31
Apolipoprotein B (g/L)	0.97 ± 0.24
Apolipoprotein A–I (g/L)	1.32 ± 0.22
Plasma triglycerides (mmol/L)	1.35 ± 0.77
*Alcohol Intake*	
None (*n*, %)	34 (24.5%)
Low, <10 g/day (*n*, %)	27 (19.4%)
Moderate, 20–30 g/day (*n*, %)	54 (38.8%)
High, >30 g/day (*n*, %)	24 (17.3%)

BMI = body mass index; SBP = systolic blood pressure; DBP = diastolic blood pressure; CAD = coronary artery disease; T2DM = type 2 diabetes mellitus; LDL-C = low density lipoprotein cholesterol; HDL-C = high density lipoprotein cholesterol.

**Table 2:** Clinical, biochemical characteristics and dietary intake for male subjects depending on alcohol intake. Data are given as mean ± SD unless stated otherwise. * Adjusted for age, smoking habits and BMI; ^a^ Significantly different from corresponding value in no alcohol intake (*p* < 0.05); ^b^ Significantly different from corresponding values of all other groups (*p* < 0.05); ^c^ Significantly different from corresponding values of all other groups (adjusted *p* < 0.05).

Characteristics	Alcohol Intake			
	No	Low	Moderate	High
	(*n* = 34)	(*n* = 27)	(*n* = 54)	(*n* = 24)
Alcohol intake (g/day)	0.0 ± 0.0	5.4 ± 2.7	19.9 ± 6.2	61.5 ± 43.0
Age (years)	34.9 ± 14.5	41.7 ± 13.4	39.9 ± 15.0	45.2 ± 10.9 ^a^
BMI (kg/m^2^)	24.6 ± 3.4	23.8 ± 2.9	24.6 ± 3.6	25.4 ± 3.8
SBP (mmHg)	127.4 ± 14.2	122.1 ± 11.1	124.4 ± 13.5	126.4 ± 15.1
DBP (mmHg)	83.0 ± 8.9	81.9 ± 9.2	80.5 ± 10.2	81.1 ± 12.6
Smoking (*n*, %)	6 (17.6%)	5 (18.5%)	12 (22.2%)	12 (50.0%) ^b^
History of CAD (*n*, %)	6 (17.6%)	3 (11.1%)	10 (18.5%)	8 (33.3%)
History of T2DM (*n*, %)	2 (5.9%)	1 (3.7%)	2 (3.7%)	1 (4.2%)
Total cholesterol (mmol/L)	5.08 ± 1.13	5.17 ± 1.11	5.33 ± 1.04	5.34 ± 0.83
LDL-C (mmol/L)	3.27 ± 1.01	3.35 ± 1.09	3.47 ± 0.98	3.43 ± 0.84
HDL-C (mmol/L)	1.20 ± 0.29	1.20 ± 0.37	1.24 ± 0.32	1.27 ± 0.27
Apolipoprotein B (g/L)	0.89 ± 0.22	0.99 ± 0.29	0.99 ± 0.22	1.02 ± 0.26
Apolipoprotein A–I (g/L)	1.34 ± 0.24	1.34 ± 0.23	1.27 ± 0.19	1.38 ± 0.22
Plasma triglycerides (mmol/L)	1.32 ± 0.78	1.32 ± 0.75	1.38 ± 0.66	1.38 ± 1.04
Fasting plasma glucose (mmol/L)	5.17 ± 0.60	5.29 ± 093	5.43 ± 1.95	5.32 ± 0.69
Fasting plasma insulin (UI/L)	8.47 ± 3.89	9.20 ± 6.07	8.25 ± 418	10.65 ± 11.59
Dietary carbohydrates intake (g/day)	278.48 ± 81.68	282.26 ± 58.33	273.60 ± 78.79	265.15 ± 68.05
Dietary fat intake (g/day)	86.95 ± 28.37	93.00 ± 24.93	87.48 ± 26.67	89.98 ± 20.95
Dietary MUFA intake (g/day)	32.96 ± 13.22	36.80 ± 10.77	33.90 ± 11.39	35.07 ± 8.93
Dietary PUFA intake (g/day)	13.61 ± 5.66	15.72 ± 4.67	13.68 ± 5.21	15.47 ± 4.48
Dietary saturated fat intake (g/day)	32.59 ± 10.72	34.02 ± 9.13	32.48 ± 10.36	32.66 ± 8.02
Energyintake (kcal/day)	2301.30 ± 586.35	2402.38 ± 481.40	2405.81 ± 608.94	2651.58 ± 599.32
∆cTG-AUC (mmol·h/L)	6.8 ± 7.0	2.8 ± 7.3	6.5 ± 5.6	7.4 ± 6.3
∆cTG-AUC (mmol·h/L) adjusted *	7.1 ± 1.8	3.1 ± 1.9 ^c^	6.5 ± 1.8	7.3 ± 2.2

BMI = body mass index; SBP = systolic blood pressure; DBP = diastolic blood pressure; CAD = coronary artery disease; T2DM = type 2 diabetes mellitus; LDL-C = low density lipoprotein cholesterol; HDL-C = high density lipoprotein cholesterol; MUFA = Mono-unsaturated fatty acids; PUFA = Poly-unsaturated fatty acids; ∆cTG-AUC = incremental area under the curve for daytime capillary triglycerides.

## 4. Discussion

Our study suggests that low alcohol intake is associated with a decrease in diurnal triglyceridemia in males after adjustment for age, BMI and smoking. Our results are in line with a recent meta-analysis and other studies on alcohol consumption, suggesting that small to moderate alcohol consumption is associated with slightly lowered plasma TG [7,8]. A recent study described that three to twenty alcoholic drinks per week were associated with lower plasma TG compared to subjects who did not drink or those who consumed >20 alcoholic drinks per week [8]. A similar trend was observed in the Danish general population who participated in the Copenhagen City Heart Study [30]. The probability value for a U-shape was significant in women (*p* = 0.006), but not in men (*p* = 0.23), although men showed a similar trend [9]. The association between low alcohol intake and a decrease in diurnal triglyceridemia may be due to genetic interactions with alcohol intake or the type of alcoholic beverages consumed, but this needs further investigation [6].

We also observed a trend that moderate and high alcohol intake was associated with increased postprandial TG after dinner and at bedtime. The lack of differences before dinner most likely reflects an efficient TG clearance mechanism in this relatively healthy population. Moreover, since alcohol consumption occurred mostly around dinner as is usually the case in a healthy population [24], the most significant effects of alcohol consumption were found after dinner. However, an animal study showed that chronic alcohol consumption increases postprandial TG without affecting fasting TG, which may suggest that the time of alcohol consumption *per se* is less important than the amount of alcohol consumed [31]. In contrast, it was recently shown in an experimental study that 0.6 g alcohol per kg body weight significantly increased postprandial TG with a prolonged postprandial lipemic response when the alcohol was consumed at breakfast [32]. Increased fasting TG without prolongation of the postprandial TG response was observed when a similar dose of alcohol was consumed the evening before (12 h before breakfast). These results suggest that alcohol consumption in the evening affects fasting TG as well.

Clear mechanistic metabolic explanations are present to explain the increase in TG upon high alcohol intake. Alcohol has been shown to stimulate the secretion of very low density lipoproteins, to reduce lipoprotein lipase activity, to stimulate adipose tissue lipolysis resulting in elevated hepatic delivery of free fatty acids and to impair the oxidation of fatty acids in the mitochondria [20,32,33,34]. In addition, the literature is consistent regarding high alcohol intake and increased TG [12,35]. For example, increased postprandial TG was observed after dinner when 40 g of alcohol was consumed daily for a period of three weeks [36]. These results are comparable with our observations, because a trend for increased cTG in males with moderate and high alcohol intake was observed after dinner and at bedtime.

Theoretically, it is possible that a bias was introduced and that some of the subjects altered their drinking habits during the study despite the fact that we asked all participants to adhere to their regular diet and drinking habits. Another limitation of the present study is the possibility of inaccuracy in the self-reported amount of alcohol intake, due to the fact that we used questionnaires and diaries [37]. However, all answered questionnaires were evaluated individually, which may have improved the validity. It should be noted that we did not observe significant differences in HDL-C among the alcohol groups although it is known that chronic alcohol consumption increases HDL-C. In the present study we have evaluated the acute effects of alcohol consumption and we do not have data on chronic use. Subjects were in a free living, non-controlled situation with different drinking habits and meals, which could have affected lipoprotein metabolism [38,39]. The majority of subjects consumed wine and beer and therefore our results cannot be extrapolated to other beverages. The impact of different beverages on TG metabolism remains uncertain, but there is some evidence that red wine has a more favourable effect on TG when compared to other types of alcoholic drinks [7,40]. Low to moderate alcohol intake has been associated with healthier lifestyle habits, as well as with the consumption of specifically red wine [41]. Red wine contains polyphenols including resveratrol, which has TG lowering effects [42]. This may explain the lower diurnal triglyceridemia associated with low alcohol intake. Moreover, it was not possible to analyse the effect of different drinking patterns on diurnal triglyceridemia with our observational study design.

## 5. Conclusions

In males, low alcohol intake was associated with lower diurnal triglyceridemia when compared to no, moderate or high alcohol intake, which was most pronounced during the evening. High alcohol intake seems to contribute to increased TG during the evening.
